# Effects of the seasonal flooding on riparian soil seed bank in the Three Gorges Reservoir Region: a case study in Shanmu River

**DOI:** 10.1186/s40064-016-2121-9

**Published:** 2016-04-21

**Authors:** Miao Zhang, Fangqing Chen, Shaohua Chen, Yajin Wang, Jianzhu Wang

**Affiliations:** Engineering Research Center of Eco-Environment in the Three Gorges Reservoir Region, Ministry of Education, China Three Gorges University, Yichang, China; International Center for Ecological Protection and Management in the Three Gorges Area, Hubei Province, China Three Gorges University, Yichang, China

**Keywords:** Species diversity, Seed density, Spatial distribution, Similarity, Three Gorges Reservoir Region

## Abstract

**Introduction:**

The water-level fluctuation in the Three Gorges Reservoir Region has changed dramatically as a result of the hydroelectric project for flood control and power generation. The riparian seasonal hydrological environment also has changed from summer flooding with winter drought to summer drought with winter flooding. The changes of riparian seed bank and vegetation were investigated to determine the effects of the seasonal flooding on the composition and spatial distribution of riparian soil seed bank and the similarity of seed bank to standing vegetation.

**Case description:**

We conducted intensive riparian soil sampling (525 samples) along altitude gradient in the Shanmu River, a tributary of the Yangzi River in the reservoir region of China. Seed bank density, species richness and composition of soil seed bank were examined using the seedling-emergence method. The seasonal hydrological conditions resulted in a decrease in species diversity and an increase in the distribution heterogeneity of the soil seed bank. The soil seed bank was composed of 48 species from 22 families and 40 genera. Most species were annual and perennial herbaceous *Polygonaceae*, *Asteraceae*, and *Poaceae*. *Rumex dentatus* was the predominant species accounting for 27.0 % of the total seeds. Diversity and composition of the seed bank changed along an altitude gradient and soil depth. Maximum species richness was found in the top soil layer at 165 m and 175 m above sea level. The mean overall seed density of the soil seed bank was 13,475.3 ind m^−2^. Density and the number of seeds increased initially and then decreased with increased altitude. Maximum seed density (22,500.2 ind m^−2^) was found at 165 m above sea level in the intermediately flooded riverbank, with the seed number accounting for 27.8 % of the total soil seed bank. Average seed density declined significantly with soil depth. The similarity of seed bank to standing vegetation was relatively high.

**Discussion and Evaluation:**

The environmental heterogeneity created by the wide range and seasonal flooding led to the changes in biodiversity and seed density along altitude gradient. The seasonal flooding also led to the increase in the similarity of seed bank to standing vegetation as their composition both degraded.

**Conclusions:**

The seasonal flooding due to the dam reshape the composition and spatial distribution of riparian soil seed bank and limit the vegetation to a grassland dominated by a few annuals and perennials in the Three Gorges Reservoir Region.

## Background

The soil seed bank provides a propagule source for the survival, dispersal, and establishment of plant populations playing an important role in vegetation regeneration, recovery and succession (Nathan and Muller-Landau 2000; Yu and Jiang 2003; Farrell et al. 2010). Composition and distribution of seeds reflect the structure of current vegetation and also determine the trend of future vegetation development (Leck 2003; Wang and Zhu 2002). Flooding is a critical ecological process influencing the riparian soil seed bank in wetland areas. It can structure the riparian soil seed bank temporally and spatially and determine seed contribution to extant vegetation (Hölzel and Otte 2001; Capon and Brock 2006). The range, duration and frequency of flooding all influence seed dispersal range and affect species composition and spatial distribution patterns of seed banks (Capon 2007; Hou et al. 2008; Osunkoya et al. 2014), and ultimately determine vegetation development (Battaglia and Collins 2006; Stroh et al. 2013; Su et al. 2012). A dramatic hydrological change can alter and degrade the riparian soil seed bank and vegetation (Hölzel and Otte 2004; Olmstead et al. 2013; Osunkoya et al. 2014). Knowledge of riparian soil seed banks and their response to the variations in water regime is crucial to understanding vegetation restoration, estimating the quality of degraded riparian ecosystems, and analyzing vegetation succession (Beatrijs and Olivier 2008; Wang et al. 2013; Nishihiro et al. 2006).

The water level in the Three Gorges Reservoir Region (TGRR) has reached 175 m above sea level following the completion of dam construction in 2009. The damming increases the flooding amplitude with up to 35 m, and switches the flooding time from summer to winter (Chen and Xie 2009; Lu et al. 2010b). These variations in water depth and flooding pattern in the reservoir are established to aid both in flood control and hydropower generation. A flooding zone about 400 km^2^ now occurs in TGRR. The reversal of flooding time, prolonged flooding duration, and new flooding zone (about 400 km^2^, transferred from terrestrial system) dramatically alter environmental conditions in the riparian zone (New and Xie 2008). Some species have disappeared from river banks as they do not adapt to the environmental changes. The composition and structure of riparian vegetation has significantly degraded (Chen and Xie 2009; Lu et al. 2010a). Wang et al. (2009) and Lu et al. (2010b) investigated the effects of the initial impoundment (155 m above sea level, in 2008) on the riparian soil seed bank in the reservoir area, and found that flooding significantly decreased species diversity and seed quantity of the soil seed bank. Meanwhile, flooding timing and depth had no effect on species diversity and seed quantity. However, there have been no additional studies since full impoundment (175 m above sea level) of the reservoir took place in 2009.

We hypothesized that increased flooding reshaped soil seed bank and caused its differentiation among sites with different elevations (flood depth and duration) in the drawdown zone of TGRR. The hypothesis was tested by analyzing the changes of species composition and seed density of soil seed bank along altitude. The objectives of this study were to (1) examine whether the characteristics of riparian soil seed bank, including composition, diversity, seed density and the similarity to standing vegetation differs between flooding zone and no-flooding zone, (2) determine whether the composition, diversity and seed density of riparian soil seed bank differ across sites with different elevations in the drawdown zone, and (3) relate the characteristics of riparian soil seed bank to the environmental changes induced by the seasonal flooding, and discuss the changing trends of vegetation to provide a scientific basis useful for riparian vegetation restoration and management.

## Methods

### Study area

**Fig. 1:**
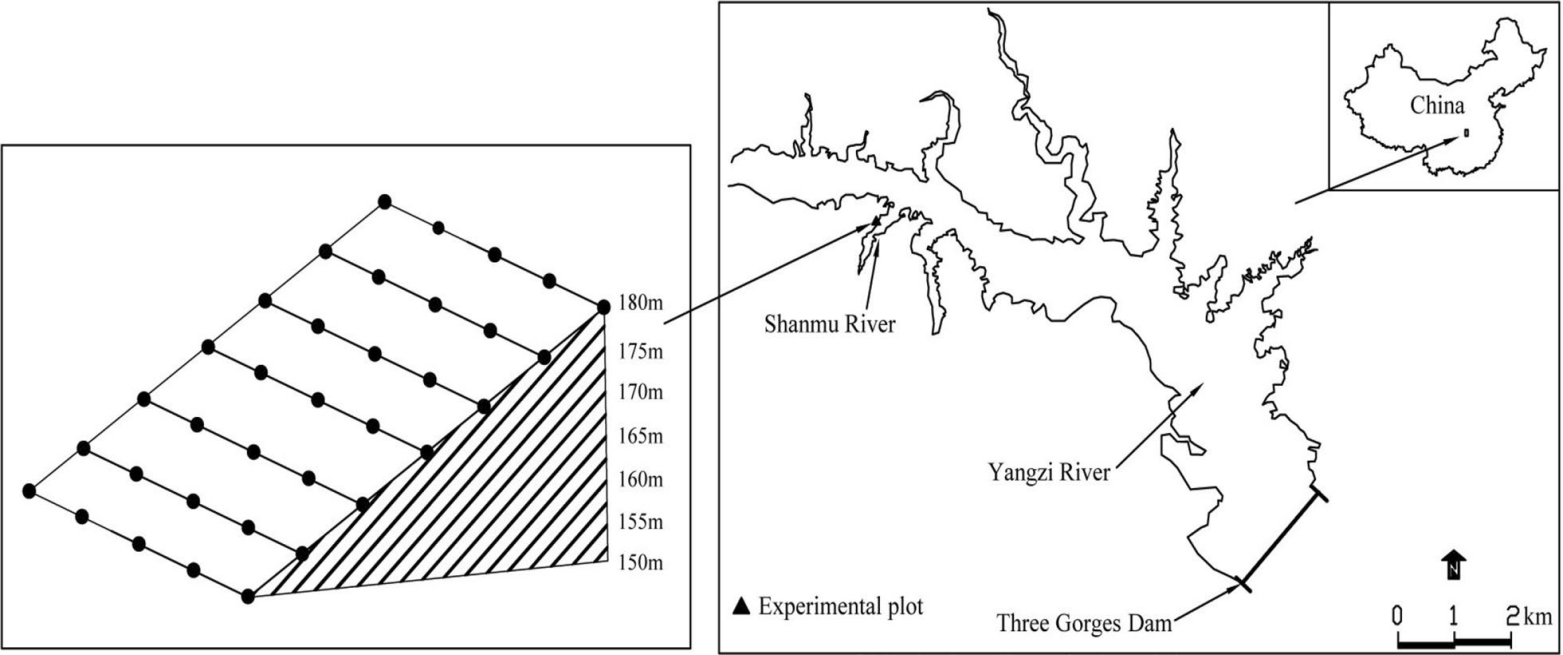
The location of experimental plot and the distribution of sample transects

The study plot was located on the riverbank of the Shanmu River (30°52′22″–30°53′17″N, 110°54′05″–110°54′28″E) (Fig. 1), a tributary of Yangzi River in Zigui County, TGRR. The regional climate is subtropical continental monsoon with four distinct seasons. Mean annual temperature is approximately 17.9 °C. Mean annual precipitation is 1100 mm with 80 % of annual precipitation occurring from April to October. The riverbank slope averaged 28°. Soil type was yellow loam with approximately 40 cm depth. Vegetation before the construction of the dam was a coniferous and deciduous broad leaf forest. This was cleared in 2007 prior to the Three Gorges Dam completion. The current vegetation in the water fluctuation zone is grassland dominated by *Cynodon dactylon*, *R. dentatus*, and *Setaria viridis*. The terrestrial vegetation above the water fluctuation zone is a mixed coniferous and deciduous broad leaf forest dominated by *Pinus massoniana* and *Quercus variabilis*.

A riverbank with typical riparian vegetation along the Shanmu River was chosen as the field experimental plot. Seven transects were established on the riverbank along an altitude gradient from 150 to 180 m at 5 m intervals covering a height of 30 m riverbank above sea level. The flooding zone is from 150 to 175 m and the non-flooding zone is from 175 to 180 m. Twenty-five quadrats (1 × 1 m^2^) were established randomly in each transect (Fig. 1). The total drawdown zone (145–175 m) experienced five cycles of submersion and subsequent exposure prior to our 2014 field tests.

### Sample collection of soil seed bank

In May 2014, filed surveys were conducted as soon as the transects were reexposed after waters subsided. Surface vegetation was removed from the quadrats. Soil was sampled randomly in each quadrat using a soil collector (8 cm diam. × 16 cm long). Each soil core was sectioned into three soil depths (0–5, 5–10, 10–15 cm) representing the topsoil, mid soil and subsoil layers, respectively. Soil from each section was wrapped in a plastic bag. A total of 525 soil samples were collected at the field experimental plot.

### Determination of soil seed bank

In laboratory, soil samples were air dried in a cool and ventilated location. Rocks, roots, plant and animal residues and other debris in soil samples were removed. Samples were gently crushed with a wooden hammer, and stones and impurities were removed using a 4 mm sieve. Species and number of seeds in samples was determined using the method of seedling-emergence (Tang et al. 1999; ter Heerdt et al. 1996; Bigwood 1988). Each soil sample was placed into a germination tray (20 cm × 15 cm × 5 cm), and spread in a 2 cm deep layer onto a 3 cm thick layer of sterile sand. The sand substrate was prepared by oven drying at 120 °C for 12 h. Germination trays were placed in a plant growth chambers, and maintained at 25 °C, 75 % RH, and a light:dark cycle of 12:12 h. Soil samples were watered to saturation daily and seedling species were identified at this time. Indentified seedlings were removed to prevent overcrowding, and the seedling number of each species was recorded. Soil was stirred to encourage seed germination in samples where few seeds germinated. The germination experiment continued until no additional seedlings appeared for 2 weeks. The germination trays with unidentified seedlings were move out growth chamber and raised in greenhouse until seedlings were identified. Seeds were classified as annual, perennial herbage, shrub or fluorescence and tree in life form for the composition comparison of soil seed bank.

### Data analysis

Seed number and the seed density of all species in each soil seed bank sample were calculated respectively. The species and seed number of each species in a soil sample included those identified by manual separation and those identified by seedling-emergence. Biodiversity indexes at each altitude and soil layer, including Shannon–Wiener diversity index, Simpson dominance index, Pielou evenness index and Margalef richness index of soil seed bank, were calculated respectively. The characteristics of soil seed bank in composition and spatial distribution were further determined by comparing species composition and life form, and the biodiversity indexes of soil seed bank among different altitudes and soil layers. A univariate analysis was used to evaluate the significance of differences in the quantitative spatial distribution of the soil seed bank. Seed density of soil sample was used as a dependent variable, and altitude gradient and soil depth were used as independent variables respectively. Duncan’s multiple range test was used to compare differences between groups when the effect of an independent variable was significant. All analyses were performed using SPSS 17.0.

The relationship of coexisting species in soil seed bank and established vegetation was classified into four types as Whipple (1978): A high percentage both in soil bank and vegetation, B high percentage in vegetation, low percentage in seed bank, C high percentage in seed bank, low percentage in vegetation, D low percentage both in soil bank and vegetation. The number of each type was used to analyze the change of similarity between the flooding zone and non-flooding zone.

## Results

### Species diversity of the soil seed bank and spatial distribution

Forty-eight plant species from 22 families and 40 genera were identified from the soil seed bank in the fluctuation zone. The dominant families include Asteraceae (12 species), Gramineae (8 species) and Polygonaceae (3 species) (Table 1). There were 15 species whose seed number each accounted for more than 1.0 % of the total soil seed bank. These species include *R. dentatus*, *Solanum nigrum* and *S. viridis*. Among them, *R. dentatus* had the maximum seed density (3626.59 ind m^−2^). Its seed number accounted for 27.0 % of the total soil seed bank. There were 12 species whose seed number each accounted for <0.01 % of the total soil seed bank. *Cyperus difformis* had the lowest seed density (1.33 ind m^−2^). In contrast, there were 32 species in 18 families and 28 genera in the riverbank non-flooding zone. Asteraceae and Gramineae had 6 and 5 species respectively. *Cyperus iria* had the maximum seed density (1608.28 ind m^−2^), accounting for 16.9 % of the total soil seed bank.

**Table 1:** Seed density of dominant species in the soil seed bank on riverbank

Riverbank	Number	Species	Family	Seed density (ind m^−2^) (mean ± SE)	Percentage (≥2.50 %)
Flooding zone	1	*Rumex dentatus* L.	Polygonaceae	3626.59 ± 699.93	27.00
	2	*Setaria viridis* (L.) Beauv	Gramineae	1447.72 ± 200.45	10.80
	3	*Setaria glauca* (L.) Beauv	Gramineae	1227.44 ± 385.89	9.10
	4	*Solanum nigrum* L.	Asteraceae	1036.36 ± 309.44	7.70
	5	*Digitaria ciliaris* (Retz.) Koel.	Gramineae	912.95 ± 233.46	6.80
	6	*Paspalum distichum* L.	Gramineae	769.64 ± 267.28	5.70
	7	*Memorialis hirta* (B1.) Wedd.	Urticaceae	559.98 ± 140.85	4.20
	8	*Gnaphalium affine* D. Don	Asteraceae	541.40 ± 120.26	4.00
	9	*Oxalis corniculata L*.	Oxalidaceae	522.82 ± 83.26	3.90
	10	*Cyperus iria* L.	Cyperaceae	444.53 ± 168.90	3.30
	11	*Phyllanthus urinaria* L.	Euphorbiaceae	395.44 ± 80.41	2.90
Non-flooding zone	1	*Cyperus iria* L.	Cyperaceae	1608.28 ± 1510.73	16.90
	2	*Memorialis hirta* (B1.) Wedd.	Urticaceae	1242.04 ± 610.96	13.10
	3	*Gnaphalium affine* D. Don	Asteraceae	899.68 ± 518.07	9.50
	4	*Oxalis corniculata* L.	Oxalidaceae	875.80 ± 93.36	9.20
	5	*Setaria viridis* (L.) Beauv	Gramineae	796.18 ± 251.46	8.40
	6	*Trigonotis peduncularis* (Trev.) Benth.	Boraginaceae	692.68 ± 160.52	7.30
	7	*Rubus phoenicolasius* Maxim.	Rosaceae	573.25 ± 214.60	6.00
	8	*Digitaria ciliaris* (Retz.) Koel.	Gramineae	501.59 ± 192.82	5.30
	9	*Artemisia argyi* H. Lév. & Vaniot	Asteraceae	445.86 ± 309.23	4.70
	10	*Viola betonicifolia* J. E. Smith	Violaceae	429.93 ± 231.58	4.50
	11	*Phyllanthus urinaria* L.	Euphorbiaceae	414.02 ± 80.21	4.40
	12	*Setaria glauca* (L.) Beauv	Gramineae	262.74 ± 128.13	2.80

Species from the soil seed bank were mainly annuals. The percentage of annual plants ranged from 61.9 to 81.8 % meanwhile perennial herbage from 14.8 to 28.6 %, shrubs and vines from 0 to 9.7 % and trees from 0 to 6.2 %, respectively (Table 2). Species diversity changed with altitude in the fluctuation zone. Altitudes 165 and 175 m had the maximum species number. Altitude 170 m had the highest evenness, diversity index and dominance index and 175 m had the highest richness index (Table 3). Species diversity also changed with soil layer. The highest richness index, diversity index and dominance index occurred at topsoil, but subsoil had the highest evenness index (Table 4).

**Table 2:** Life form composition of the soil seed bank at different altitudes on riverbank

Life form	150 m	155 m	160 m	165 m	170 m	175 m	180 m
A (%)	81.8	61.9	71.4	71.9	81.5	68.8	64.5
B (%)	18.2	28.6	21.4	18.8	14.8	18.8	25.8
C (%)	–	9.5	7.1	6.3	3.7	6.2	9.7
D (%)	–	–		3.1	–	6.2	–

A, annual; B, perennial herbage; C, shrub or fluorescence; D, tree; -, no occurrence

**Table 3:** The changes of diversity indices of the soil seed bank with altitude gradient

Altitude (m)	Species number	Margalef index	Simpson index	Shannon–Wiener index	Pielou index
150	22	2.122	0.866	2.399	0.776
155	21	1.955	0.800	2.045	0.672
160	28	2.483	0.828	2.284	0.686
165	32	2.665	0.842	2.256	0.651
170	27	2.251	0.900	2.595	0.787
175	32	2.727	0.868	2.428	0.701
180	31	2.786	0.911	2.694	0.785

**Table 4:** Diversity indices of the soil seed bank in different soil layers

Types	Soil layer (cm)	Species number	Margalef index	Simpson index	Shannon–Wiener index	Pielou index
Flooding zone	0–5	42	3.296	0.891	2.654	0.710
	5–10	34	2.851	0.859	2.447	0.694
	10–15	30	2.705	0.886	2.582	0.759
Non-flooding zone	0–5	26	2.444	0.901	2.540	0.780
	5–10	18	1.837	0.878	2.416	0.836
	10–15	22	2.279	0.906	2.594	0.839

### Quantitative characteristics of soil seed bank and spatial distribution

Average seed density of the soil seed bank was 13,475.3 ind m^−2^ in the fluctuation zone, which was greater than that in the non-flooding zone. The quantitative distribution of the riparian soil seed bank changed significantly with altitude gradient in the fluctuation zone (P < 0.01) (Table 5). The maximum seed density (22,500.2 ± 6998.4 ind m^−2^) occurred at 165 m, where the seed number accounted for 27.8 % of the total soil seed bank. Seed density within the soil seed banks decreased with increasing and decreasing altitude. The seed density at the lower fluctuation zone was lower than that at the upper fluctuation zone. The minimum seed density (3972.9 ± 409.4 ind m^−2^) occurred at 150 m.

**Table 5:** Change of seed density of seed bank with altitude gradient

Riverbank	Altitude (m)	Seed density (ind m^−2^) (mean ± SE)	Percentage (%)
Flooding zone	150	3972.9 ± 409.4d	4.9
	155	5637.0 ± 811.1d	7.0
	160	10,660.9 ± 1959.7c	13.2
	165	22,500.2 ± 6998.4a	27.8
	170	20,812.1 ± 3295.1ab	25.7
	175	17,269.1 ± 3537.5b	21.4
Non-flooding zone	180	9562.1 ± 2066.7cd	–
F value; Sig.		4.628; 0.000**	–

The letters following the seed density indicate the level of difference among altitudes at P < 0.05

** The mean difference is significant at the 0.01 level

The quantitative distribution of the soil seed bank differed significantly among soil layers (P < 0.01) (Table 6). Most seeds were distributed in the 0–5 cm deep topsoil. The average seed density of the soil seed bank in the topsoil was 8426.2 ind m^−2^, and the seed number accounted for 62.5 % of the total seed bank. Soil seed bank density decreased with soil depth declining to 3540.3 and 1505.4 ind m^−2^ at soil depths of 5–10 and 10–15 cm, respectively. The vertical distribution of the soil seed bank at 180 m the non-flooding zone showed a pattern similar to that of the fluctuation zone. However, the percentage differences among the topsoil, middle soil and subsoil on the non-flooding zone were lower than that of the fluctuation zone.

**Table 6:** Changes of seed density in soil seed bank with soil layers

Riverbank	Soil layer (cm)	Seed density (ind m^−2^) (mean ± SE)	Percentage (%)
Flooding zone	0–5	8426.2 ± 1243.4a	62.5
	5–10	3540.3 ± 753.2b	26.3
	10–15	1505.4 ± 266.8b	11.2
F value; Sig.		17.375; 0.000**	–
Non-flooding zone	0–5	5501.6 ± 1527.1a	57.5
	5–10	2054.1 ± 698.8b	21.5
	10–15	2006.4 ± 120.5b	21.0
F value; Sig.		4.251; 0.000**	–

Different letters following the seed density indicate significant difference among soil layers at P < 0.05

** The mean difference is significant at the 0.01 level

**Fig. 2:**
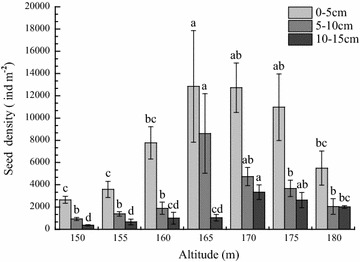
Changes in seed density of the soil seed bank with altitude and soil depth. *Different letters* above columns indicate significant difference among soil layers at P < 0.05

**Fig. 3:**
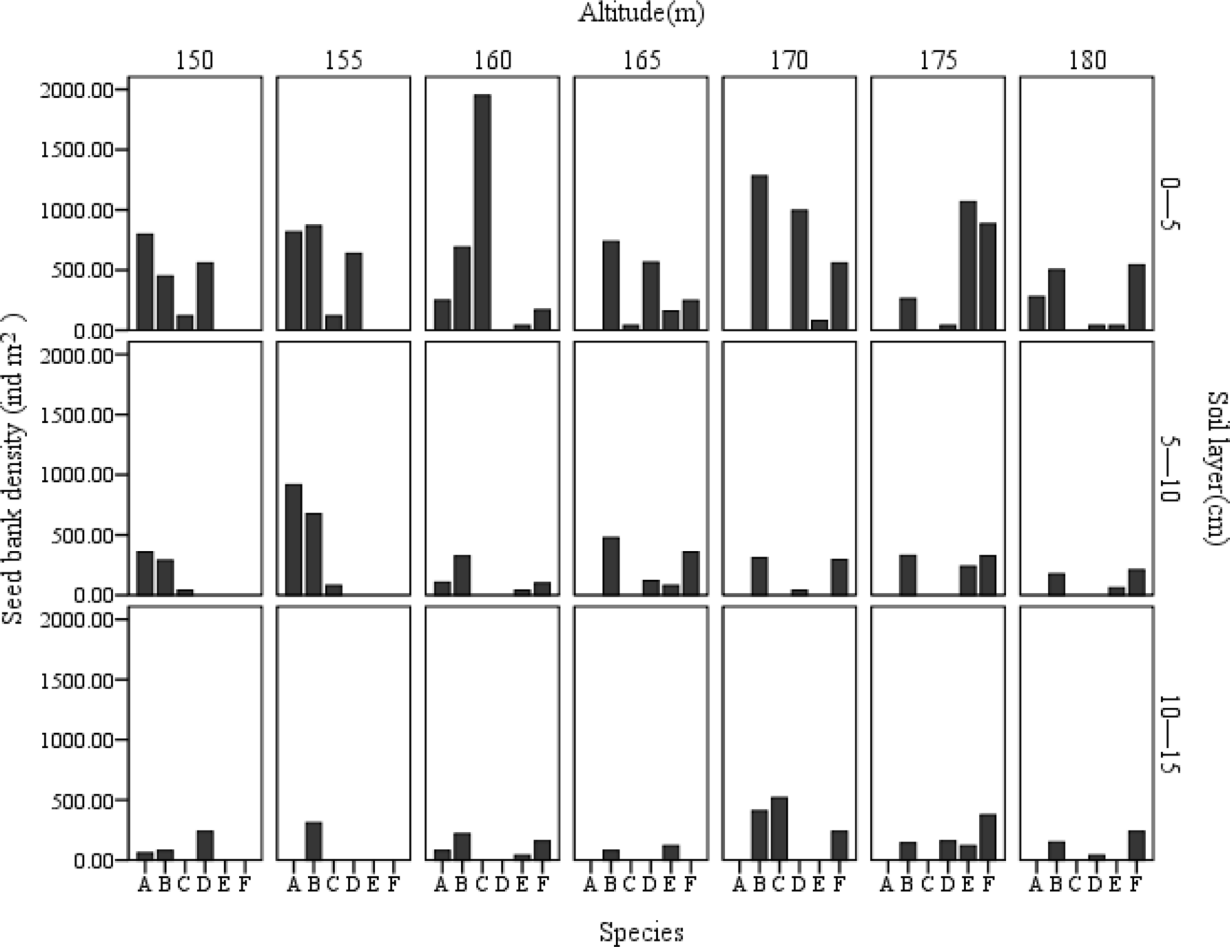
The spatial distribution of dominant species in soil seed bank. A, *Cynodon dactylon*; B, *Setaria viridis*; C, *Mazus japonicus*; D, *Bidens pilosa*; E, *Perilla frutescens*; F, *Gnaphalium affine*

The vertical distribution pattern of the riparian soil seed bank varied with altitude. The seed density in topsoil was greater than that in the mid soil and subsoil. The maximum seed density of the soil seed bank in topsoil, middle soil and subsoil appeared at 165 and 170 m respectively (Fig. 2). However, each dominant species had its spatial distribution pattern (Fig. 3). *Mazus japonicus* was restricted to the middle and low flooding zone, so were *Gnaphalium affine* to the middle and upon flooding zone. *S. viridis*, meanwhile, distributed on all flooding zone.

### The similarity of soil seed bank to established vegetation

In the flooding zone, there were 30 species coexisting both in soil and vegetation, 33 and 18 species only in vegetation and soil, respectively. The similarity index of seed bank to standing vegetation was about 0.531. In the non-flooding zone, by contrast, 19 species occurred both in soil and vegetation, 36 and 12 species only in vegetation and soil seed bank with relative low similarity index 0.223 (Table 7).

**Table 7:** Similarity of riparian soil seed bank to standing vegetation

Riverbank	Total species in vegetation	Total species in seed bank	Species only in vegetation	Species only in seed bank	Coexisting species	Similarity index
Flooding zone	63	48	33	18	30	0.531
Non-flooding zone	55	31	36	12	19	0.223

**Fig. 4:**
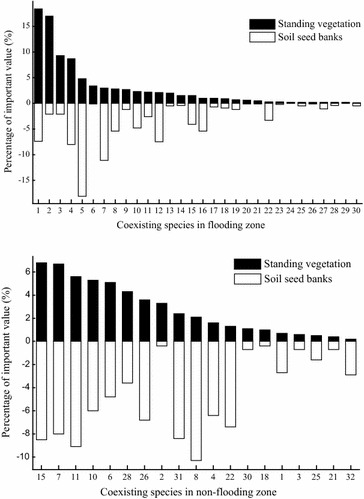
The percentage of important value accounting for by the coexisting species in seed bank and vegetation on the riverbank. 1, *Setaria glauca*; 2, *Cynodon dactylon;* 3, *Bidens frondosa*; 4, *Digitaria ciliaris*; 5, *Rumex dentatus*; 6, *Artemisia argyi*; *7, Setaria viridis*; *8, Hyrtanandra*; 9, *Polygonum hydropiper*; 10, *Phyllanthus urinaria*; 11, *Cyperus iria*; 12, *Solanum nigrum*; 13, *Xanthium sibiricum*; 14, *Bidens pilosa*; 15, *Gnaphalium affine*; 16, *Paspalum distichum*; 17, *Mazus japonicus*; 18, *Eclipta prostrata*; 19, *Acalypha australis*; 20, *Youngia japonica*; 21, *Phytolacca acinosa*; 22, *Trigonotis peduncularis*; 23, *Aeschynomene indica*; 24, *Polygonum aviculare*; 25, *Magnolia multiflora*; 26, *Rubus phoenicolasius;* 27, *Stellaria media*; 28, *Viola betonicifolia*; 29, *Senecio scandens;* 30, *Glochidion wilsonii*; 31, *Oxalis corniculata*; 32, *Cyperus rotundus*

The relationship of coexisting species in soil seed bank and established vegetation differentiated on the Shanmu River riverbank. There were 10 type A species, 6 type B species, 6 type C species and 8 type D species representing by *Setaria glauca*, *Artemisia argyi*, *Trigonotis peduncularis* and *Senecio scandens*, respectively, on the flooding zone. However, only 7 type A species, 4 type B species, 5 type C species and 3 type D species appeared on the non-flooding zone. There were more coexisting and dominant species in seed bank and vegetation on the flooding zone which increased their similarity (Fig. 4).

## Discussion

Flooding has significant influences on seed sources which further determine species composition of the riparian soil seed bank (Leck 2003; Peterson and Baldwin 2004). Nicol et al. (2003) studied the influence of water regime on seed bank composition and found that seed compositions were correlated with the water regime and independent of the initial seed bank composition irrespective of differences in the initial seed bank composition. The construction of the Three Gorges Dam altered the flooding regime in the reservoir from summer submersion-winter exposure to summer exposure-winter submersion to improve flood control and power generation. Seasonal timing of inundation stressed riparian plant growth and flowering (Greet et al. 2013). The average duration of flooding along the altitude gradients from 150 to 175 m ranged from 240 to 30 days (Wang and Gao 2010). With the seasonal flooding, only a small number of annuals and a few flood-tolerant perennials were able to complete their life cycle and produce seeds during the limited exposure time, especially at the lower altitude. Our field investigation indicated that the standing riparian vegetation along the Shanmu River was composed of 63 species. Among these species, seeds of 33 species were not present in the riparian soil seed bank. The species composition of the riparian soil seed bank along the Shanmu River was simplified and mainly consisted of selected annuals accounting for 68.8 % of the total species number.

The change of species composition in the soil seed bank differed among different impoundment periods in the TGRR. During the primary impoundment, species composition in the soil seed bank was relatively complex and included the seeds produced by standing vegetation. The survival of seeds produced by vegetation before impoundment was dominated by annuals and the dominant species in this vegetation included perennials, annuals and shrubs (Wang et al. 2009; Lu et al. 2010b). Our results indicated that species composition of the soil seed bank was further simplified after a long period of water level fluctuation. Many pre-dam riparian shrub and perennial species disappeared from the riparian soil seed bank. The soil seed bank was dominated by some annuals and a few flooding-tolerant perennials.

Flooding also affects seed dispersing and deposing from standing and surrounding vegetation, and influenced species distribution in the riparian soil seed bank (Boedeltje et al. 2004; Xiao et al. 2011; Alves Pagotto et al. 2011; Hayashi et al. 2012). The timing of seed dispersal and river flow interact to determine the annual composition of species cohorts (Dixon 2003). Species groups segregated on the basis of exposure length of sediments to air, rate of drawdown, and the water depth (Nicol et al. 2003; Xu et al. 2007). The maximal species richness of riparian seed bank appeared where vegetation had the most species (Calcada et al. 2015). Species diversity of the riparian soil seed bank varied with the altitude gradient along the Shanmu River. The maximum diversity appeared at the intermediately flooded zone, while the least diversity was found at the lower riverbank. Intermediate diversity occurred at the upper fluctuation zone. Exposure and fluctuation times in the middle upper fluctuation zone coincided with seed maturity of many plant species allowing this zone a relatively greater opportunity to receive air and water-dispersed seeds.

The diversity distribution of the soil seed bank differed among soil layers. The top soil layer had the highest richness and dominance index and subsoil had the highest evenness. A potential mechanism causing this trend is because all of the dispersed seeds were first deposited on the top soil layer. The soil seed bank on the terrestrial zone had the same spatial distribution as the fluctuation zone. But the difference between topsoil and subsoil was relatively small, indicating that water-level fluctuations and submersion did not favor seed deposition into deeper soil layers.

The intensity, duration, and frequency of water-level fluctuations significantly influence distribution of the riparian soil seed bank (Weiterova 2008; Liu et al. 2006). The season and time that seeds, dispersed by wind and flow, fall on the riverbank changed with altitude because water-level fluctuations affect submersion and exposure times of riverbank areas (Peterson and Baldwin 2004). This also resulted in spatial heterogeneity in quantitative composition of plants within the soil seed bank (Yuan et al. 2007; James et al. 2007; Nielsen et al. 2012). Under the influence of this seasonal flooding, the submersion and exposure seasons of the riverbank changed with altitude gradient and shaped the quantitative spatial distribution of the riparian soil seed bank (Wang et al. 2009). Wang et al. (2010) and Lu et al. (2010b) found that the primary impoundment in TGRR led to the decrease in seed density of the soil seed bank in the fluctuation zone. The average seed density was about 12,667 and 4578 ind m^−2^, respectively. However, differences among altitudes were not significant. Our results indicated that a new quantitative distribution pattern of soil seed bank was formed after a lengthy water-level fluctuation period in TGRR. The average seed density in the soil seed bank on the Shanmu River riverbank was 16,517.0 ind m^2^. The highest seed bank density and total seed number appeared at 165 m (the middle upper fluctuation zone). This exposed area and fluctuation time coincided with the summer and autumn seed maturation periods of many plant species, providing significant opportunities to receive air and water-dispersed seeds. In contrast, the lower fluctuation zone was only exposed and fluctuated during summer. The short exposure time limited seed availability and reduced the seed density of the soil seed bank to a low level. The upper fluctuation zone was exposed for the longest time, and had ample time to receive seeds dispersed by air. However, the water fluctuations at this location in winter were at an inappropriate time for seed spread by water. Thus, the seed density and seed number of the soil seed bank at the lower and upper flooding zone were relatively low.

The environmental heterogeneity of soil also contributed to quantitative distribution variation of the soil seed bank. Anoxia resulting from submersion and drought stress can both decrease seed vitality (Riddin and Adams 2009). The seasonal flooding created moisture, temperature, and oxygen soil conditions at the middle upper riverbank favorable for seed survival. In contrast, the lower riverbank was stressed by lengthy submersion, and the upper riverbank was stressed by prolonged drought.

Water-level fluctuations caused spatial differences in seed density among soil layers in the soil seed bank. Seed density of the soil seed bank decreased with soil depth as seeds in the subsoil generally came from the infiltration of seeds in the top soil layer. The quantity of infiltrated seeds decreased with soil depth. Initial seed quantity in the topsoil and soil depth both determined seed distribution in the subsoil. Decreases in altitude and soil depth caused the decrease of seed viability as the soil oxygen, moisture and temperature became less favorable (Chen and Xie 2009; Carta et al. 2013).

The composition and biodiversity of seed bank determine the similarity of seed bank to established vegetation (Wilson et al. 1993; Xiao et al. 2011). James et al. (2007) suggested that the similarity increased when soil seed bank was mainly composed of annual herbs hereabouts, but decreased when mainly composed of perennial herbs, shrub and trees. There were significant differences in species composition and dominant species between the soil seed bank and the surrounding vegetation during the primary impoundment in the TGRR (Wang et al. 2009; Lu et al. 2010b). The similarity of seed bank to vegetation was relatively low, but increased as a result of the seasonal flooding (Wang et al. 2010). Our results suggested that long-term seasonal flooding further induced the composition of seed bank simple, which increased the similarity index of seed bank to established vegetation.

The soil seed bank may contribute to plant community dynamics following disturbance (Plassmann et al. 2009; Liu et al. 2009). The seasonal flooding caused the degradation of soil seed bank and vegetation on the river bank in the TGRR. Most perennials and woody plants disappeared from seed bank and vegetation, and the soil seed bank was mainly composed of annuals and a few flooding-tolerant perennials. Lu et al. (2010b) investigated the effects of initial impoundment on the riparian soil seed bank and suggested that low species compositional similarity to established vegetation and the dominance of annual plants limited the efficacy of the soil seed bank to restore pre-dam vegetation in the drawdown zone of the TGRR. Our study indicates that the differences in species composition and dominant species between the soil seed bank and surrounding vegetation decreased, implying the vegetation composition and structure gradually stabilized after a long period of water-level fluctuation. The composition of the soil seed bank and vegetation included annuals, perennials and shrubs and vines that differed from the pre-dam riparian soil seed bank and vegetation. The dominant species of the soil seed bank, including selected annuals and several submersion-tolerant perennials, such as *C. dactylon* and *P. paspaloides*, was related to the standing vegetation.

## Conclusion

Under the influence of seasonal flooding, the soil seed bank degraded and was mainly composed of annuals differing significantly from the pre-dam soil seed bank and standing vegetation. The environmental heterogeneity created by the wide range and seasonal flooding led to the changes in biodiversity and seed density along altitude gradient. Maximum species richness and seed density occurred at 165 m above sea level. The seasonal flooding also increased the similarity of seed bank to established vegetation as both of the composition degraded. The dominant species of the soil seed bank, including selected annuals and several submersion-tolerant perennials, such as *C. dactylon* and *P. paspaloides*, was related to the standing vegetation. It is concluded that the seasonal flooding due to the damming determine the composition and spatial distribution of seed bank and limit the riparian vegetation to a grassland dominated by annuals and perennials in the TGRR.
